# Burnout Syndrome and Logotherapy: Logotherapy as Useful Conceptual Framework for Explanation and Prevention of Burnout

**DOI:** 10.3389/fpsyt.2019.00382

**Published:** 2019-06-14

**Authors:** Norbert Riethof, Petr Bob

**Affiliations:** ^1^Department of Psychiatry, First Faculty of Medicine, Charles University, Prague, Czechia; ^2^Central European Institute of Technology, Faculty of Medicine, Masaryk University, Brno, Czechia

**Keywords:** burnout syndrome, logotherapy, existential vacuum, existential fulfilment, existential meaning

## Abstract

Burnout syndrome is a state of total exhaustion related to work conditions and stress from work. Recent findings suggest that logotherapy and the concepts of existential meaning and life fulfilment could provide a useful framework for explaining and potentially preventing burnout. This review article summarizes and reflects current knowledge concerning the relation between burnout syndrome and existential vacuum as a potential correlate. It also explores the risks of burnout and the need of better definition of this condition including more precise diagnostic criteria and internationally recognized measurement tools. Intensified research on relations between burnout and lack of existential fulfilment and meaning could help with future prevention and intervention design.

## Introduction

“In order to burn out, a person needs to have been on fire at one time.”

—Ayala Pines

A purpose of this review article is to link Viktor Frankl’s concept of existential vacuum to burnout syndrome. It will address the fact that despite the increasing number of recent articles about burnout syndrome, very few studies have explored the connection of burnout with loss of meaning in one’s work and life. Recent findings suggest burnout syndrome is in fact a form of existential vacuum described by Viktor Frankl (1) as part of his logotherapy with loss of both life fulfilment and existential meaning as crucial elements (2–4). In addition, this article will describe a need for better understanding of this syndrome as a potentially widely established diagnostic category and how to distinguish it from other related diseases like depression or stress-related disorders. Based on the recent findings, the potential risk of unrecognized and untreated burnout syndrome is very high on personal, professional and social levels (5–8). However, it is necessary to investigate this disease much more extensively and establish a diagnostic category including standardized, internationally recognized and valid methods for the differential diagnostics; otherwise it will not make any sense to try and estimate the percentage of people who are in danger of burnout (9, 10).

Freudenberger (11) in his paper called “Staff burnout” described the symptoms of burnout for the first time. Before him, the term was used in Graham Greene’s novel “Burnt-out case” from 1960. Freudenberger observed volunteers for aid organizations and noticed that these people after only few months of initial enthusiasm became “burned out” (12–14). Burnout syndrome was later defined by Maslach and Jackson as a set of three symptoms: 1) *emotional exhaustion*, 2) *depersonalization and cynicism* and 3) feelings of *inefficiency or lack of accomplishment* (15, 16). This three-dimensional model has been widely accepted as a conceptual framework for burnout syndrome, and WHO used it in its definition of burnout in the latest version of the *International Classification of Diseases*. What is advantageous about this model is positioning of stress experience of an individual into a social context and conceptualizing self and others (17). Sonneck (18) expanded this definition of burnout and used the term “vital instability” to add other burnout symptoms like depression, dysphoria, excitability, inhibition, anxiety, restlessness, despair and irritability.

The danger of burnout syndrome in any profession is that a person can appear fully functioning at work but emotionally drained, depersonalized and even suicidal. The people around them usually do not notice any problems for a very long time, because a person with burnout very often feels guilty about their attitude and behavior and they have a tendency to hide their true feelings (19). People with burnout syndrome feel they are trapped in their situation, imprisoned and with a feeling they are not able to get out of it. They often feel like victims of the situation or given circumstances. Extreme stress work conditions can lead to life-threatening states. In Japan in 1978, there was a special term defined for an “overwork death”—it is called Karōshi. Karōshi is a sudden death resulting from unbearable states of exhaustion or starvation diet and caused by stroke (60% of victims) or heart attack (10%) (20). Amongst common causes for the development of burnout syndrome, we can usually find very demanding or frustrating job (very often in helping professions) and a subsequent series of negative personal changes in attitude and behavior (21–23).

The need for better diagnostic tools and strategies for prevention are even more urgent considering potential psychosocial and psychosomatic risk factors for professionals in healthcare, education, social work, management etc. Burnout can result in severe problems with both physical and mental health, and it can lead to depression or addictive disorders. Stressful situations may also affect physiological processes, and thus burnout syndrome may lead to metabolic disturbances (6–8). If a person with burnout has also symptoms of depression, drug dependency and/or despair, it can become a very dangerous state with suicidal tendencies (2). However, the recognition of burnout syndrome on the international level is still problematic (24). Burnout’s unclear status also limits the possibility of disability claims and access to treatment; therefore, the need for more precise and objective assessment has become critical (17, 25). WHO in its 11th version of the *International Classification of Diseases* defined burnout syndrome according to Maslach’s original definition but narrowed it down specifically to the occupational context with a recommendation it should not be applied to other areas of life. The Diagnostic and Statistical Manual of Mental Disorders should provide more precise criteria in the future so it will be possible to diagnose burnout as an occupational disease (25).

For the years 1947 to 2018, a systematic literature research was done in electronic databases (PubMed, Science Direct, EMBASE, PsycINFO and Springer Link). The important inclusion criteria were burnout, logotherapy, therapeutic intervention, stress, depression and treatment outcome.

## Burnout Syndrome

Burnout usually occurs in the context of work and/or demanding social relationships. Because of the diagnostic unclarity of burnout syndrome, burnout is often explained and associated with existing diagnostic categories like stress-related disorders or a specific type of depression. Some authors even suggest that we should redefine burnout to job-related depression and thus be better able to detect unhealthy states at the workplace with an available disease category (9). In addition, cultural tendencies for avoiding stigma from mental disease might be present, and the term burnout can be used as a socially more acceptable description for depressive episodes (26). However, the reductionism of burnout syndrome to specific and individual reaction to stress or work overload seems to be misleading. It is important to recognize that one of the differentiating qualities of burnout is a crisis of meaning and values, and therefore it would be incorrect to focus on just exhaustion and its connection to work overload or stress (17).

In addition, there is an ongoing discussion on who can be affected by this syndrome. Originally, this syndrome was detected mainly amongst professionals in healthcare. Later it was extended to other social work positions. Several authors, however, suggest that burnout is not restricted only to healthcare and social work. Pines and Aronson (27) regard burnout as a symptom that can be seen in any professional or even nonprofessional work. Also, some jobs nowadays require very high levels of customer service and burnout has become relevant for occupations that require intense level of personal and emotional contact as well (28). Recent studies amongst various working groups and in various countries have suggested that each person regardless of age or profession (and not restricted to helping professions) can be affected by burnout syndrome, especially social professions with high emotional load, i.e. teachers, pastors, trainers, physicians, palliate care specialists, lawyers, managers, policemen, but also women on maternity leave or even unemployed people (29–32).

According to Maslach and Jackson (15) symptoms of burnout syndrome can be grouped into three main categories: 1) *emotional exhaustion*, in which demanding work involves experiencing difficult emotions that consume a person’s energy; 2) *depersonalization and cynicism*, in which people “protect” themselves by disengaging from the relationships and difficult emotions related to work; and 3) feelings of *inefficiency or incompetence*, in which people no longer consider themselves as able to achieve or accomplish something. There is an agreement amongst researchers that burnout needs development over time and it does not happen instantly. It is usually a slow process that may take years, rather than months. However, the understanding of the precise development of burnout and how many stages are included are still not unified (33). There are also different versions of stage order, while there is a common agreement that burnout development involves relatively distinct phases (34).

Freudenberger and North (12, 122–156) have divided the process of development of burnout syndrome into 12 phases. These phases are outlined in **Table 1**.

**Table 1:** Twelve phases of burnout syndrome by Freudenberger and North.

1. The Compulsion to Prove Oneself; demonstrating self-worth obsessively; showing enthusiasm; accepting responsibility easily. 2. Working Harder; reinforced efforts; an inability to switch off and relax from work. 3. Neglecting Needs; problems with sleep; eating disorders; lower level of social interaction. 4. Displacement of Conflicts; problems are repressed; feelings of threat, panic and nervousness. 5. Revision of Values; values are reinterpreted; friends and family neglected; hobbies seem irrelevant; work is the only focus. 6. Denial of Emerging Problems; intolerance; perceiving collaborators as stupid, lazy, demanding or undisciplined; social contacts harder; signs of cynicism and aggression; problems are viewed as caused by time pressure and work, not because of life changes. 7. Withdrawal; social life very small or nonexistent; need to feel relief from stress; abuse of alcohol/drugs. 8. Odd Behavioral Changes; visible changes in behavior; friends and family concerned. 9. Depersonalization; seeing neither self nor others as valuable; not being able to perceive own needs. 10. Inner Emptiness; feeling empty inside and to overcome this, look for activity such as overeating, sex, alcohol or drugs; activities are often exaggerated. 11. Depression; feeling lost and unsure, exhausted; future feels bleak and dark. 12. Burnout; can include total mental and physical collapse; time for full medical attention.

Maslach et al. (16) reframed these stages and simplified them to four phases, phase 4 being considered as a heavy disorder:

1. *Idealism and overload:* trying to “prove” something to oneself or others, neglecting personal needs
2. *Emotional and physical exhaustion:* repression of needs, change of values, repression of conflicts
3. *Dehumanization as a protection:* unhappiness, discontent with oneself is dominating, personal connection is withdrawn
4. *Terminal phase:* syndrome of unwillingness and loathing syndrome, disgust against oneself, others, work, finally against everything and finally breakdown (professional resignation, illness, suicidality).

According to some authors there are certain clusters of causes for explaining symptomatology of burnout syndrome. Längle and Künz (32) have suggested three clusters. The first one is *individual psychological explanation* describing psychological and personality risk factors like perfectionism, idealism, narcissism, goal-orientation and depressive tendencies. The second cluster is *social psychological explanation* emphasizing experiences like bullying, suppression, mobbing or very overloading, difficult work or service (trauma units at hospitals, managerial positions etc.) And the third one is *organizational framework* with situational circumstances like conflict of roles, no or little feedback, dysfunctional teamwork, too little autonomy at work, too high expectations but too little information or quantity of work being simply too much. A combination of high ambitions and high efforts over a long period of time, low satisfaction from work (poor outcomes) and conditions at work that induce stress seems to be causing burnout with high probability (19). Demerouti et al. (35) also found that factors like low job security, lack of feedback, lack of resources and high job demands have a potential to lead to two main components of burnout—exhaustion and disengagement. Other research results indicate that frequent causes of burnout amongst teachers are circumstances like high expectations from own performance, too heavy workload, unclear job roles, inadequate reward, decreased autonomy and demanding tasks (4). On the contrary, the best predictor to describe a good working environment is subjectively perceived autonomy and open and supportive communication. For some burnout researchers, the opposite of burnout has been identified as work engagement (17, 28, 29). And engagement can be then measured as opposite scores on the three scales of the Maslach Burnout Inventory (MBI) questionnaire (17).

There is an ongoing debate on what the relation is between burnout and depression: whether burnout is an accelerating factor for depression or vice versa or maybe if those two categories are the same thing, which would make burnout a mental illness (17). Empirical research has suggested that the two constructs are indeed separate entities, the main differentiator being that burnout is job related, while depression is more general and context-free. However, some studies confirm that burnout and depression are not solely independent. It seems that depressive and burnout symptomatology share similar “qualitative” characteristics, especially in the more severe forms and also in the final stages of burnout and in individuals that are more vulnerable and have little satisfaction from their work (19). But we are still not able to say that burnout and depression are the same mental illness (17). The relation between burnout syndrome and depression is high; some symptoms are the same—especially in the final stages of burnout (loss of motivation and energy, feeling of meaninglessness)—and thus some researchers suggest burnout is a type of depression (36). According to Bianchi et al. (37) the current state of research is not able to clearly distinguish burnout from depression, especially in the final stage of the burnout process when the symptoms are very similar to clinical depression. In addition, there are many different forms of depression and we should not compare burnout to unspecified depressive symptoms but rather investigate the correlation between burnout and atypical forms of depression. However, one of the main differentiators is the willingness to live in people with burnout in contrast to patients with depression (except the very last stage of burnout syndrome) and burnout as work or activities related disorder (38).

Burnout has been also often mistaken for stress, because stress is an integral part of burnout. There is no burnout syndrome without stress. Stress from work seems to be present at least at the beginning of burnout syndrome development in each burnout case. Similarly as with depression, stress and burnout have a lot of symptoms in common. However, stress symptoms are more physical and not so emotional. The opposite applies for burnout. And stress is not the main cause of burnout development, although it can accelerate its progression (33). Stress usually leads to hyperactivity and feelings of urgency. Burnout rather leads to feelings of hopelessness and helplessness. When we are having stress, we tend to overreact on the emotional level, but when we experience burnout, our emotions are much weaker (34). Work today brings along a lot of stress, not only satisfaction or a certain socioeconomic status. To control the job environment is very difficult; that is why job stress is usually higher than stress from personal life. But only stress from work itself does not cause burnout. If the work environment provides professionals with positive feedback, they can function at high productivity level over a long period of time without burnout (19).

### Measurement of Burnout Syndrome

According to some authors, researchers have reached a consensus on how to measure burnout (4), and they consider MBI as a common standard and widely used and accepted instrument, while others argue that the theoretical concept of burnout syndrome is still not clear (39) and we must consider or invent some other or new tools for measurement of burnout than MBI (37). Numerous other instruments have been invented over the last decades—Burnout Measure (BM), Shirom–Melamed Burnout Inventory (SMBM), Oldenburg Burnout Inventory (OLBI) or Copenhagen Burnout Inventory (CBI). In a systematic literature search from 2015 about research comparing burnout and depression, it was found that MBI was used in 78%, SMBM in 14%, BM in 6% and OLBI in 2% of the studies (37). However, a lot of symptoms of burnout are measured through self-reporting scales (rather than researching directly other features of behavior or health associated with burnout) which represents another critical constraint for measurement (17).

#### Maslach Burnout Inventory

Maslach Burnout Inventory (MBI) was first published in 1981 by Maslach and Jackson and revised in 1986 (15, 40). Twenty-two questions of this measurement tool are focused on three main components of burnout syndrome (nine items for emotional exhaustion, five items for depersonalization and eight items for personal accomplishment), and they can be assessed individually. Later in the 1990s, a more general version of MBI was created called MBI-GS (General Survey). The MBI-GS was based on the original MBI, but the term “recipients” was removed from all questions and a few new questions were added. Thanks to deletion of the word “recipients” the MBI-GS exhaustion scale is more generic (41). Also, the three dimensions have been renamed—from emotional exhaustion to exhaustion, from depersonalization to cynicism and from personal accomplishment to professional efficacy. Until 1998, the MBI was used in more than 90% of all empirical burnout studies in the world and MBI was seen as the dominating and almost only measurement of burnout (42). However, some researchers are critical to this new MBI-GS because according to them it is still not clear what exactly does the new instrument measure? “We have not been able to find any (new) definition of burnout in connection with the presentation of the new questionnaire” (39).

#### Burnout Measure

The second most commonly used measure of burnout is the questionnaire called BM—Burnout Measure (26, 39, 43). BM is a newer version of the questionnaires “The Tedium Scale” and “Tedium Measure” by Ayala Pines who created it together with Elliot Aronson (43). The instrument consists of 21 questions scored on a seven-point Likert scale from Never (1) to Always (7) (44). It was translated to several languages, and it is being used in Germany, France, the Netherlands and Spain. According to Pines (27), burnout syndrome is a final stadium of the exhaustion process (attrition) through which highly motivated individuals lose their enthusiasm. This tool was created for use across a whole range of different occupations, including unemployed people. The author has considered three main attributes of burnout—psychic (mental), physical and emotional, yet this instrument is still a one-dimensional tool. It does not work with three factors, but only with a single final score. BM has a positive correlation with the subsequent terminations of employment (*r* = 0.58). Although it is a one-dimension inventory with three subscales, it correlates well with emotional exhaustion scale from MBI (*r* = 0.70) and depersonalization (*r* = 0.50), not so well with personal achievement scale (*r* = 0.25) (45).

## Burnout Within a Framework of Logotherapy

Very few researchers go beyond established categories of burnout symptom and suggest other origins of burnout than prolonged stress or work overload. A promising approach is to explain burnout syndrome within a concept of logotherapy formulated by Viktor Emil Frankl. From the point of view of logotherapy, the burnout is an affliction generated by loss of existential meaning. Frankl suggested that the existential meaning is fundamentally important to mental health, and this finding led Frankl (46) to the formulation of the concept *existential vacuum*. Frankl defined the term existential vacuum as a loss of life interests and a lack of initiative and proactiveness, which can lead to deep feelings of meaninglessness (47). These two aspects of existential vacuum can be explained in more detail with the following description related to burnout (2):

1. *Losing interest*—nothing is interesting anymore in burnout syndrome because of exhaustion and by being overstressed by problems which we do not have time to resolve.
2. *Lack of initiative*—person is not motivated to do anything, growing apathy, nothing seems to have value, nothing is attractive, passive behavior, feeling of helplessness.

Frankl defined in his anthropology three dimensions of human existence (46)—physical sphere, mental sphere and noetic sphere. Symptoms of burnout can manifest on all three spheres (2, 48)—see **Table 2**.

**Table 2:** Burnout manifestation on three Viktor Frankl’s dimensions of human existence.

1. *Physical sphere:* physical weakness, sleeping disorders, susceptibility to diseases, reduced immunity, cardiovascular diseases, muscular and nape tensions, etc. 2. *Mental (psychological) sphere:* emotional exhaustion, indifference, apathy, hopelessness, sadness, dysphoria, loss of optimism, loss of happiness, increase in irritability. 3. *Noetic sphere:* disrespect to self and to the world, lower self-esteem, retreat from relationships and connection with the outer world, loss of spiritual orientation, doubts about value of life.

Burnout syndrome can be explained through this Frankl’s model as a deficit primarily in the noetic sphere. There is a danger of feeling existentially frustrated, when people try to satisfy only their inner needs and drives (lust, need for power etc.) and do not pay attention to noetic (spiritual) dimension. When living a life like that (only satisfying “psychodynamic” needs), one can develop symptoms of existential vacuum (1). Two dominant symptoms of existential vacuum—feelings of emptiness (apathy) and meaninglessness (loss of interest)—are similar to later stages in the development of burnout syndrome (2). Burnout can be seen as a manifestation of an existential vacuum. However, the main difference to Frankl’s explanation of existential vacuum is the absence of boredom and apathy in burnout syndrome. They are included in Frankl’s definition of the existential vacuum, but they are merely the consequence of other symptoms that are present in burnout people (2). Also, people suffering from an existential vacuum seem to have highly developed two main characteristics of burnout—depersonalization and emotional exhaustion (14).

Origins of burnout syndrome can be better understood as a lack of existential meaning. A person achieves existential meaning through the feelings of inner fulfilment. Inner fulfilment gives a person the power and persistence to go through fatigue and exhaustion, especially when they also maintain a feeling of freedom and high self-esteem (49). People with burnout syndrome experience a deficit of inner fulfilment; we might also say they seek in their lives things that are not truly fulfilling and in which they rather experience feelings of “I *must* have it” or “I *must* do it.” From a perspective of logotherapy and existential analysis, people with burnout syndrome perform activities and they are engaged in tasks and duties towards which they do not feel an existential meaning. They do not experience their personal fulfilment. Instead, they experience burnout which can be defined as a “disorder of well-being, caused by a deficit of fulfilment” (2).

The decisive difference of Frankl’s anthropology to the traditional three-dimensional concept (body, psyche and mind/spirit) is the recognition of a dynamic interactive nature of the three spheres. According to Frankl, the noetic (spiritual) sphere is taking a stance towards and sometimes against (independently on) the psyche and the body. Thanks to this independence of noetic dimension we as people have freedom to decide and be active in the world. The noetic dimension gives us the possibility to look at ourselves objectively (from a distance) and independently on our current somatic or psychic states. It also enables us to engage in meaningful activities that go beyond pure satisfaction of our physical or psychological needs. Frankl named these fundamentally human capacities “self-distance” and “self-transcendence.” The self-transcendence competence (also called spiritual) enables a person to interact with the world beyond the self, and it is being seen as essentially human, giving the ultimate meaning to life (1, 2, 50).

### Measurement of Existential Meaning and Life Fulfilment

Several attempts were made to measure the noetic or “existential” abilities and the concept of fulfilment in life according to Frankl’s theory. Frankl (51) introduced the definition of meaning as “a possibility on the background of reality,” and he also stated that meaning is fulfilled by realizing values. Längle (52) elaborated on these concepts of meaning by adding to them a more personal and subjective aspect, and he developed the following definition of existential meaning: “Meaning is the most valuable possibility in the actual situation.” The term “actual situation” is a concept taken from existential philosophy describing the actual circumstances in which a person is embedded. Taking this definition as a basis, Längle (53) derived the Meaning-Finding Method with four steps reflecting four “personal competencies for existence” (in brackets):

1. Realistic perception (self-distance)
2. Free emotionality (self-transcendence)
3. Decision-making ability (freedom)
4. Acting (responsibility)

Längle et al. (54) operationalized his method by constructing the Existence Scale (ES) with 46 items, 4 subscales and 3 generalized factors and designed it as a self-rating questionnaire measuring personal abilities to reach existential meaning and life fulfilment. The test is based on Frankl’s anthropology and his existential analysis (55), and it reflects four stages of development of a meaningful and fulfilling existence of a person according to Längle’s variation of existential analysis (53). It follows a standardized self-rating procedure on a six-point Likert scale, ranging from “fully disagree” to “fully agree.” It has 8 items related to self-distance, 14 items related to self-transcendence, 11 items related to freedom and 13 items related to responsibility.

Another similar questionnaire (based on the Existence Scale) is the Existential Fulfilment Scale (EFS), which took three existential concepts—self-acceptance, self-actualization and self-transcendence—and considers them as three distinct dimensions of existential fulfilment (56). The EFS consists of 15 items, 5 items for each dimension, measured on a five-point Likert scale, running from 0 to 4, meaning “not at all” to “fully” relevant to me (5). Amongst some other instruments that are in use in relation to the concept of “existential vacuum” is the “Purpose-in-Life-Test” (“PIL”) by Crumbaugh and Maholick (57) and the “Logo-Test” by Lukas (58) which measures the accomplishment of meaning and existential frustration (47).

## Relation of Lack of Existential Fulfilment and Burnout Syndrome

Existential and humanistic psychology offered the perspective that life (existential) fulfilment is a prerequisite to healthy human functioning. Frankl (50) introduced the potential relation between his psychology of meaning (logotherapy) and burnout symptomatology. In contrast to his term “existential vacuum” we can put the opposite concept “existential fulfilment” which refers to experiencing life as being full of meaning and purpose and which induces psychological well-being (5). Existential fulfilment can be defined as “the life-purpose that aims at doing full justice to the nature of human existence” (56). By existential fulfilment we mean feeling of fulfilment in a whole life, where work represents only one part. But life and work cannot be separated; it is one entity (4). If people are not able to find meaning in their lives, they may be vulnerable to the development of burnout (59).

In today’s world people seek meaning and fulfilment of their needs on the noetic dimension (according to Frankl’s anthropology), no longer in religious and community life, but very often at work. This is a potential source of their frustration and disappointment because their expectations about what their jobs can provide to their well-being were too high and unrealistic (4, 27, 42, 60). This factor might also contribute to the rise of burnout, along with an increasing work stress and higher demands from all organizational and social “stakeholders” (clients, patients, managers, shareholders etc.) However, at the beginning of burnout development we usually see very intense experience of meaningful life, work, existence etc. We encounter the loss of existential meaning at the end of the process when a person’s efforts failed, the barriers and demands were too high, the circumstances were incredibly unfavorable and people feel their endeavor crashed completely. In this context, Maslach and Leiter (28) offered a fairly radical definition of burnout syndrome: “Burnout is the index of the dislocation between what people are and what they have to do. It represents an erosion in value, dignity, spirit, and will—an erosion of the human soul. It is a malady that spreads gradually and continuously over time, putting people into a downward spiral from which it’s hard to recover.”

According to Längle (2) burnout syndrome is related to the loss (or deficit) of existential meaning. Loss of meaning in life is caused by the fact that people do things not because of the things themselves but because of some other (usually external) reason and motivation (i.e. career, money, social influence etc.) which provides the feeling of a so-called apparent meaning. There is a lack of “truth” in one’s activity (in what one is doing) and a presence of “foreign” motivation. This apparent meaning has no meaningful content. But people can fall into burnout also because they are experiencing too much meaning and they are not able to decide between various priorities, which leads to exhaustion. Längle differentiates between real existential meaning and “pseudo-meaning.” From the point of view of his existential analysis burnout is a disorder of psychological well-being which comes from a lack of inner fulfilment. This inner fulfilment is a result of life devoted to something where a person realizes subjectively important (perceived) value. Burnout syndrome is the last stage of a long-lasting state of experiencing working without felt value in what a person does. In people with burnout syndrome the intention to work is not directed towards the actual job, nor the task itself. It is a subjective intention that leads them away from what they do (for career, for reward, for recognition, respect, self-value, social acceptance, fulfilling the duties, power, influence, to get things done in order to be free from them etc.) They are not interested in the job itself. The motivation is leading away from the baseline of action. It results in “pseudo-motivation,” and psychologically they are not really occupied with what they do. There is a gap between subjective motive and real doing. The burnout people are not interested in the content of their work (32).

Burnout appears not through valuable contents (which provide existential meaning) but by formal, foreign “pseudo-motivation” which leads to “pseudo-turning” towards the work activity. The person does not feel attracted by the value of the work, but they feel pushed by it. The work has to be done for something else, and this results in the feelings “I must do it” or “there is no other way.” There remains an interesting question of how these “pseudo-motivations” and “pseudo-meanings” can be so strong that people will end up in such an unhealthy state for a very long time. These motives must have deeper roots—they are not just opinions or thoughts. They might be psychically deeply anchored needs (32). Nevertheless, the best prevention from burnout is to experience fulfilment at work and doing a job with pleasure and interest. In such a case, the risk of ending up in a burnout is very low (2). But the feelings of fulfilment should be differentiated from enthusiasm, idealism, feeling of success etc. Enthusiasm may lead to burnout, because enthusiasm is often not realistic; it is idealizing the situation and it is expecting a success, usually nothing else. However, a person can experience inner fulfilment even if they are not successful, when the activity itself is perceived as good and meaningful, but it is not “crowned” with a success. Burnout people approach their work in a utilitarian way—as a means to achieving their goals—not as something of inherent value and meaning. But as a consequence, other areas of life (leisure time, private life) will become affected by this overall feeling of meaninglessness, and this feeling will finally consume a person’s life in its totality (2).

Many professionals show a very high degree of commitment to work these days. Also, they identify with their profession and organization so much that every mistake is being perceived as something traumatic, to the point of almost losing meaning of life when they fail (19). In today’s secularized world, the meaning of life is usually no longer provided by religion, but it is being replaced by work. And people try to use work as an alternative “source” for feelings of life fulfilment and being significant (61). Especially individuals with idealistic tendencies work hard in order to give more meaning to their existence. This tendency to idealization and “black-and-white” perception has been also described in the concept of splitting which may relate to burnout syndrome. Splitting was described by Kernberg (62), and it is characterised by switching between contradictory perceptions towards the same object based on pleasurable good rewarding and painful bad punishing experiences (63). In the context of burnout syndrome we observe people having a lot of (perhaps naïve) enthusiasm and idealism towards work at the beginning and in contrast big disappointment and disillusionment later (“all good” or “all bad” attitude). Some studies also suggest a relationship between burnout and other personality traits like introversion or narcissistic tendencies (64).

Several research studies (5, 65, 66) have suggested that a degree of existential fulfilment might be related to a development of a burnout syndrome. A low level of existential fulfilment and low perception of existential meaning correspond with high burnout scores. Up to this date, not so many studies about burnout have tried to measure existential fulfilment and meaning and have identified them as a determinant of burnout (65, 67). Experience of existential meaning out of work can help to prevent and protect from burnout as some recent research findings amongst physicians suggested (68). Current prevention and intervention initiatives against burnout are aimed mainly on the objective work conditions or individual stress relief, and that does not seem to be enough in order to target the core cause of burnout. Neither recreation and relaxation techniques nor stress management programs themselves can fill the void of inner meaning and fulfilling experiences (2). We need to focus on increasing the sense of meaning. In relation to meaninglessness, Pines (69) sees burnout as a failed attempt to get existential meaning out of work, especially in today’s world when religious aspects of life are more and more declining and people are focused—perhaps unrealistically—on the meaning of work. On the other hand, up to this date there is a limited evidence of the effectiveness of therapies addressing burnout syndrome, with the exception of cognitive-behavioral therapy (CBT) (70).

## Conclusion

Some research studies have shown that existential fulfilment and existential meaning are associated with burnout dimensions. Burnout emerges out of the experience of meaninglessness. A low level of existential fulfilment corresponds with high burnout scores. The contribution of the lack of existential fulfilment to the development of burnout has been confirmed in several studies (3–4, 5, 65, 66). However, further investigation of the frequency, determinants, potential risks and treatment of burnout is still needed. There is also a need to further investigate possible correlations between various personality traits or tendencies (i.e., splitting, narcissism etc.) and burnout syndrome. Such findings could be used in screening programs in which these personal tendencies would represent a possible key risk factor for the development of burnout syndrome. In addition, more research is needed in the area of measuring life fulfilment on one side and feelings of meaninglessness on the other and their correlations to burnout. These findings could be used as a key focus in the prevention programs and in future intervention design.

Despite the agreement on the core definition of burnout (i.e., state of total exhaustion of one’s resources), after decades of research considerable confusion exists about the theoretical concept of burnout and number and nature of other dimensions involved and how to measure them on an international level with comparable tools (10). For example, some instruments not only assess burnout on the individual level but take into account the impact of organizational conditions as well (45). Other descriptions of burnout include stress factors, and for some researchers there is still a question if burnout syndrome can be seen as a psychiatric diagnosis or whether it is rather a set of generic stress-induced depressive symptoms (8). Nevertheless, based on the recent findings, the potential risk of unrecognized and untreated burnout syndrome is very high on a personal level (health issues, risk of total exhaustion, breakdown or even suicide), on relationships (cynical attitude towards oneself and to others) or professional effectiveness (i.e., potential threat to the quality of patient/client care, absenteeism, reduction of productivity, increased number of errors caused by burnout employees or decreased quality of decision making etc.) (4, 5, 19).

Because of the number of different operationalizations of the burnout construct, there is also a clear need for better definition of burnout as a diagnostic category and for international standardization of measurement tools and standardized methods of the differential diagnostics (10, 37).

## Author Contributions

Both authors contributed to conception of the work, analysis, interpretation, drafting the work and its revision. Both authors approved the final version.

## Funding

This work was supported by Charles University grants (Progress and SVV).

## Conflict of Interest Statement

The authors declare that the research was conducted in the absence of any commercial or financial relationships that could be construed as a potential conflict of interest.
